# 

**Published:** 1903-05-15

**Authors:** 


### ANNOUNCEMENTS.

THE Indiana State Board of Examiners meets June 9th, at Indianapolis.

THE West Virginia State Board of Examiners meets June 3rd to 5th, at Charleston, W. Va.

THE Michigan State Board of Dental Examiners meets May 12th to 14th, at Grand Rapids. For particulars address C. J. Gray, Secretary, Petoskey, Mich.

DR. S. Straith has opened an office in Detroit for the exclusive application of nitrous oxide gas for anesthetic purposes, not only for extracting teeth but for other surgical purposes.

THE Dental Department of the Columbus (Ohio) Medical University graduated sixty-two dentists at the commencement, April 16th. Fifty-eight physicians and ten pharmacists were graduated at the same time.

Dr. L. P. Bethel will move his home and the editorial offices of the Dental Summary to Columbus, Ohio, in September. This is made necessary because of the increasing duties connected with the office of Dean in the college.

The Missouri State Society which meets in Kansas City, May 19th to 21st, announces nine papers and fifteen clinics on live topics by some of the best practitioners in the West. This promises a good and profitable meeting to any who may be so situated that they can attend.

The Kentucky State Dental Association will hold its meeting May 25th to 27th, at Bowling Green, this year. This will be a good meeting and affords a good opportunity of visiting Mammoth Cave. Address Dr. C. R. Shacklette, 636 Fourth Ave., Louisville, for particulars.

The Pennsylvania Board of Dental Examiners will meet June 9th to 12th simultaneously, in Philadelphia and Pittsburg, for passing on the acquirements of candidates wishing to practice in that State. Persons wishing preliminary information should communicate with the "Dental Council," Harrisburg, Penn.

As a result of the exposure of the fraudulent work done by dental colleges in this country, the highest court in the

Kingdom of Saxony, Germany, has decided that dentists holding diplomas from such colleges shall not be allowed to use a doctor's degree on their signs or to publish it in any way. This practically puts them out of business.

THE Ohio State Board of Dental Examiners will meet June 30th and July 1st and 2nd at the Hartman Hotel, Columbus, Ohio, for the purpose of examining candidates for registration. All who expect to take the examination should notify Dr. H. C. Brown, Secretary, 112 East Broad Street, Columbus, not later than June 20th. Dr. Brown will also give any information necessary on application.

THE Detroit Dental Society celebrated its twenty-first anniversary, April 18th, with a series of interesting clinics at the Dental College in the afternoon and a banquet at the St. Clair Hotel in the evening. In the matter of attendance this has been the banner year of the society, and the work done by it compares favorably with that of any other year. Much of the credit belongs to Dr. Hogarth, the president, who has labored earnestly to make his administration successful.

THE Northern Ohio Dental Association holds its forty-fourth annual meeting at Cleveland on the second day of June this year. This is one of the oldest and best dental societies in existence. Several famous men have enriched our literature through this organization, and under its present vigorous management we may expect more good work from it. Its program for this meeting promises twelve papers, fourteen clinics, sixteen exhibits and a poem entitled "Von Letel Poy" by Ohio's dental poet laureate, Dr. J. F. Siddell, of Oberlin.

